# On the path-based entanglement purification model in online quantum networks

**DOI:** 10.1038/s41598-026-70573-8

**Published:** 2026-09-08

**Authors:** Joy Halder, Nayeem Ahmed, Hilal Sultan Duranoglu Tunc, Riccardo Bassoli, Frank H. P. Fitzek, Gerhard P. Fettweis

**Affiliations:** 1 Vodafone Chair Mobile Communications Systems, Technische Universität Dresden, Dresden, Germany; 2 Deutsche Telekom Chair of Communication Networks, Technische Universität Dresden, Dresden, Germany; 3 Quantum Communication Networks Research Group, Technische Universität Dresden, Dresden, Germany; 4 Centre for Tactile Internet with Human-in-the-Loop, Technische Universität Dresden, Dresden, Germany; 5 Barkhausen Institut, Dresden, Germany

**Keywords:** Mathematics and computing, Physics

## Abstract

In this study, we propose a resource allocation model for end-to-end (E2E) fidelity-guaranteed entanglement distribution considering novel path-based entanglement purification (PEP) model for online quantum networks. The proposed PEP model is developed by extending the existing link-based entanglement purification (LEP) model. A heuristic framework is designed for both the PEP and LEP models, consisting of two stages: (i) an E2E entanglement purification stage based on the PEP (or LEP) model, and (ii) a resource allocation stage. The resource allocation stage employs an integer linear programming (ILP) formulation for small-scale quantum networks with a limited number of requests, and a greedy-based heuristic for large-scale networks with higher request volumes. This heuristic algorithm incorporates entanglement distribution probability (EDP), entanglement swapping probability (ESP), and fidelity metrics to ensure feasibility under noisy intermediate-scale quantum (NISQ) constraints. An online traffic model is considered, where requests arrive according to a Poisson process, each with a fixed execution time and a variable deadline. A time-slotted operation framework is adopted for the quantum network. In each Bell-pair generation window, Bell pairs are generated and distributed between adjacent nodes. The duration of these windows is constrained by the decoherence time of the Bell pairs. Extensive simulations are conducted on the square-grid quantum networks of varying dimensions and parameter ranges, including different ranges of EDP, ESP, and fidelity values. The number of requests and capacity of edges (in terms of quantum channels) are also varied. Across all simulation setups, the proposed PEP model consistently outperforms the LEP model in terms of the blocking ratio (*BR*).

## Introduction

With recent advances in quantum computing, information processing, sensing, and quantum key distribution, quantum networks have attracted significant attention as enabling technology for communication among distributed quantum devices^1–4^. The key functionalities of a quantum network are: (i) generate Bell pairs and distribute them between adjacent node pairs in the network, and (ii) establish an end-to-end (E2E) entanglement channel between two remote nodes through entanglement swapping operations^5–7^. However, Bell pair generation, distribution, and entanglement swapping are inherently probabilistic processes with no counterparts in classical computing^8–10^. Consequently, routing and E2E entanglement distribution in quantum networks must leverage quantum-mechanical properties in conjunction with classical communication to enable the successful transmission of quantum states between distant nodes^11,12^.

The maximally entangled Bell pairs are represented as $\frac{1}{\sqrt{2}} (\vert {00} \rangle \pm \vert {11} \rangle )$ and $\frac{1}{\sqrt{2}} (\vert {01} \rangle \pm \vert {10} \rangle )$^8,9^, where $\vert {0} \rangle \equiv \vert {H} \rangle$ and $\vert {1} \rangle \equiv \vert {V} \rangle$ denote the horizontal and vertical photon polarizations, respectively^13^. A set of Bell pairs is distributed between adjacent node pairs with a certain entanglement distribution probability (EDP) value. If this operation succeeds, an entanglement channel is established between the corresponding node pair. However, these Bell pairs are susceptible to various noise sources that degrade their fidelity. The fidelity of a Bell pair is expressed as a real number in the range [0, 1], representing the closeness of a quantum state to its ideal (or expected) state^8,14^, where a value of 1 indicates a perfect state. Quantum memories are used to store these distributed Bell pairs and preserve their fidelity. Nevertheless, these memories can maintain the fidelity of a Bell pair only for a limited duration, known as quantum decoherence time^15,16^. Once this time is exceeded, the Bell pairs are discarded due to decoherence noise. The E2E entanglement distribution between two remote nodes is established by performing entanglement swapping operations at the intermediate node(s). This swapping process is also probabilistic in nature with a certain value of the entanglement swapping probability (ESP) and depends on several external factors^11,17^. If the swapping operation succeeds at each intermediate node, an E2E entanglement channel is established between the end nodes. However, each entanglement swapping operation reduces the fidelity of the established entanglement channel. Specifically, if the fidelity values of two Bell pairs are $F_1$ and $F_2$, then the resulting fidelity after swapping becomes $F_1 F_2$. Since $F_1, F_2 < 1$, the overall E2E fidelity decreases exponentially with increasing number of swapping operations. Nevertheless, the E2E fidelity of any entanglement distribution must remain above a predefined threshold value to ensure the quality of quantum transmission. Performing entanglement purification is a potential approach to improve the fidelity of Bell pairs that degrade due to swapping operations^18–21^.

The entanglement purification process consumes two Bell pairs and produces a single Bell pair with improved fidelity. Similarly to EDP and ESP operations, the purification process is also probabilistic in nature. Suppose a bit-flip channel model, if the input Bell pairs have fidelity values $F_1$ and $F_2$, the probability of successful purification is given by $F_1 F_2 + (1 - F_1)(1 - F_2)$^18,22^. This purification process is generally classified into two categories based on the fidelity of the distributed Bell pairs between adjacent node pairs: symmetric and non-symmetric purification operations. When all Bell pairs between adjacent nodes possess identical fidelity values, the symmetric purification model is applied; otherwise, the non-symmetric model is employed^18^. In the case of symmetric purification along a path in which Bell pairs exhibit varying fidelity levels, the resulting E2E fidelity depends on the sequence in which entanglement purification and swapping operations are performed^23^.

In recent years, several studies have addressed the problem of E2E distribution and purification of Bell pairs along routing paths in quantum networks. However, entanglement-based routing introduces several inherent challenges that must be considered when tackling this problem. First, qubits are extremely sensitive to environmental interactions, which induce noise and degrade their quantum coherence^1^. This degradation impacts qubit generation, transmission, storage, and measurement. Second, the no-cloning theorem prohibits qubits from being duplicated or stored analogously to classical information, ruling out the use of classical transmission techniques. Third, entanglement generation between adjacent nodes is inherently probabilistic and prone to failure^7,15^. Fourth, the fidelity of distributed Bell pairs decreases with each successive entanglement-swapping operation, leading to a decline in the overall quality of service (QoS). Finally, the lifetime of successfully generated entanglement is limited by decoherence, which restricts the temporal validity of each Bell pair to very short durations^5,7^. Collectively, these factors require careful consideration in the design of routing protocols to enable robust entanglement distribution in quantum networks. A number of existing works considered the distribution of entangled pairs in quantum networks. A post-selected, noise-resilient entanglement generation approach is presented for large quantum networks^24^ which has been exploited in the QKD network. A link-based entanglement purification protocol is presented for bit-flip channel model in quantum networks^22,23^. Selection of the route in a communication network is often handled using the segment-based approach considering the parameters of the link^25^. This idea is also explored in a segmented quantum network to maximize network throughput and improve resource utilization^26^. The path with the known link parameters, e.g. EDP, fidelity values of the Bell pairs, can be segmented and the resource allocation problem can be formulated as a segment routing problem where the objective is to maximize the fidelity of the end-to-end entanglement while minimizing resource usage. Single/Multiple path selection is another aspect while designing efficient routing strategies in the resource-constrained classical networks^27,28^ or in the quantum networks^9,16,29^ The routing problem can be resolved by selecting one or multiple concurrent path edge-disjoint paths^9,16^. For the concurrent multipath-based approach, more than one edge-disjoint paths are computed, and E2E entanglement distributions are established, maintaining fidelity constraint for each of these paths. The cross-layer optimization technique^30^ can also be adopted in quantum networks for balancing a set of parameters like entangled channels, variable decoherence time etc. with performance objectives e.g. blocking ratio and E2E fidelity. However, no existing work addresses all the parameters of quantum networks such as EDP, ESP, and entanglement purification while designing E2E fidelity-aware routing. This leads to a fundamental question: how can an efficient model be developed for routing, E2E entanglement distribution, and entanglement purification in heterogeneous quantum networks such that fidelity-aware routing is achieved while optimizing Bell-pair utilization?

This study considers the aforementioned unsolved problem and proposes a novel path-based entanglement purification model (abbreviated as PEP) for online quantum networks. This model is developed based on the results obtained from the existing link-based entanglement purification (LEP) model^23^ for a given route and E2E fidelity threshold. The key contributions of this work are described below.

1. The path-based entanglement purification (PEP) model is proposed for online and time-slotted quantum networks. The PEP model considers noisy intermediate-scale quantum (NISQ) constraints such as EDP, ESP, and time-slotted quantum operations.
2. A resource allocation model is developed for E2E entanglement distribution and purification using LEP and PEP models for online quantum networks. For small problem size, an ILP based formulation is designed, and a greedy heuristic-based algorithm is developed for large problem size.
3. The performance of the existing LEP and proposed PEP models is compared in terms of blocking ratio (*BR*). Experiments are conducted on a heterogeneous quantum network with node-specific ESPs and varying EDP and fidelity values across edges, using an online request model for dynamic resource allocation.
4. The experimental results ensure that the proposed PEP model outperforms the LEP model in terms of *BR* in all experimental scenarios considered.

The remainder of this paper is organized as follows. First, a theoretical background on quantum networks and entanglement purification operations is provided. Next the existing LEP model and the proposed PEP model as its extension are described. Two corresponding optimization models based on ILP and greedy heuristic are developed. Finally, the experimental results obtained from these models are analyzed and the direction of future work is discussed.

## System network model

The quantum network is defined as a system consisting of several quantum devices to communicate, process, store, and sense quantum systems (e.g., qubits). It is represented as a graph *G*(*V*, *E*) with *V* as the set of quantum devices or nodes, and these quantum nodes are connected by a quantum edge set *E*^5,7,10^. In this work, a centralized quantum network architecture is considered, where information about the Bell pairs distributed throughout the network and their fidelity values is known to each node^15^. A brief description of different components of a quantum network is provided below.

### Quantum nodes

A quantum node includes quantum repeaters and quantum routers. It acts as a quantum information processing device capable of generating, manipulating, transmitting, and storing qubits^5,10^. Quantum repeaters are used to extend the distance of the entanglement distribution by performing the entanglement swapping operation. The routers in a node connect all adjacent nodes to it. Quantum repeaters and quantum routers act together to interconnect numerous nodes in quantum networks. Quantum repeaters are assumed to be trusted^31^. Each adjacent node pair consists of a number of quantum memory units to perform the entanglement swapping operation^15,16^. Each node *u* has a fixed ESP $Q_u \in [0,1]$.Each node is capable of performing entanglement purification operations in the Bell pairs successfully distributed between adjacent nodes with it^23^.

### Quantum edges

A quantum edge connects two adjacent nodes through a number of quantum channels or entanglement links. A successfully generated entanglement link is in the form of a maximally entangled 2-qubit state or Bell pair^5,15^. The Bell pairs distributed in any edge (*u*, *v*) have the following two properties: (i) the distributed Bell pairs have a fixed EDP $P_{uv} \in [0,1]$, and (ii) The distributed Bell pairs have a fidelity value $F_{uv}\in [0,1]$.

### Quantum memories

Each quantum node consists of a set of quantum memories in each direction of its adjacent node(s)^15,16^. These memories held the generated entanglement channels with a cut-off decoherence time $t_c$^15^ and the qubit stored in that memory maintains its fidelity value for $t_c$ duration of time^15^. A qubit is discarded after the interval $t_c$ due to decoherence. In the centralized network model, it is assumed that all memories in the network are assumed to be updated simultaneously^5,15^.

In this work, it is assumed that the same number (*M*) number of Bell pairs are distributed between each adjacent node pair in each window because of the same noise model in each edge. However, in case of a quantum network with the heterogeneous noise model, the probability of error model may differ in edges. In that case, the number of distributed Bell pairs may differ based on the noise model and the EDP. For example, a continuous-time Markov chain model can be adopted for computing the number of Bell-pair to be distributed per edge in a quantum network^32^.

### Time-slotted quantum operation

In this work, the time-slotted quantum operation is considered, where the operational time is divided into multiple Bell pair generation windows^7,15^. Each window has a fixed duration of $t_c$, which is the decoherence time of the quantum memories^15^. Let *W* be the set of such windows. At the beginning of each window *w ∈ W*, *M* the number of Bell pairs is distributed between each adjacent node pairs (*u*, *v*) with EDP $P_{uv}$. Let $M^{w}_{uv}$ denote the number of successfully distributed Bell pairs on edge (*u*, *v*) during window *w*. As $P_{uv} \le 1$, $M^{w}_{uv} \le M$. It is assumed that ESP $Q_u$ for any node *u*, EDP $P_{uv}$, and fidelity $F_{uv}$ for each node pair (*u*, *v*) remain the same in each window.

**Fig. 1:**
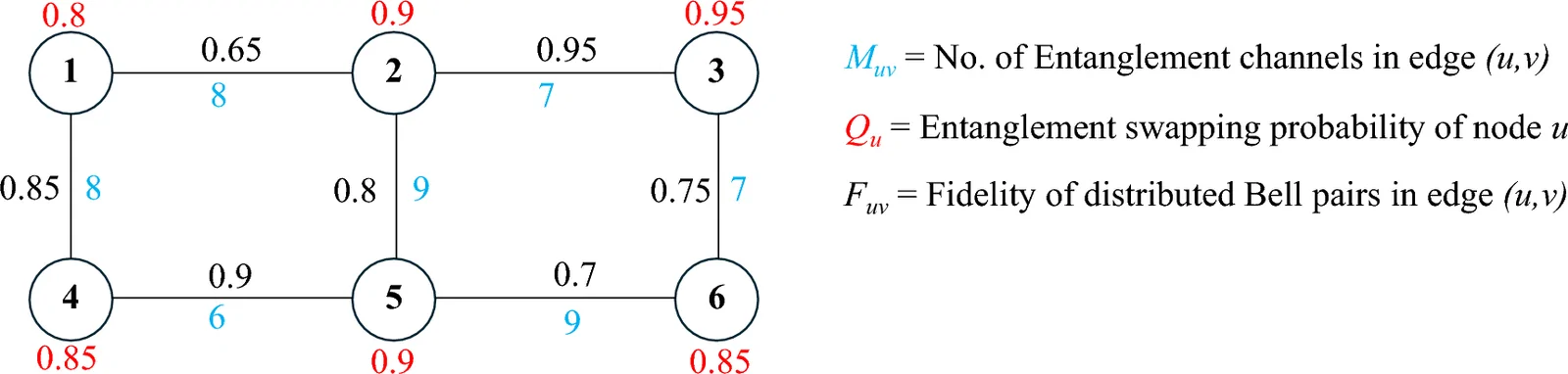
Six node and seven edge sample quantum network.

Figure 1 represents a quantum network with six nodes and seven edges considering a symmetric purification model where (i) each node has a fixed ESP (marked in red) and (ii) Bell pairs on each edge have a fixed fidelity value (marked in black). 10 Bell pairs are distributed between each node pair (*M*=10) with EDP *P*=0.8. The number of successfully distributed Bell pairs between each node pair is shown in blue.

### Request model

In case of the online quantum network model, the request set *R* is generated dynamically. Each incoming request *r ∈ R* is represented by the tuple $\langle s^r, d^r, b^r, t^r, a^r, h^r, \partial ^r \rangle$, where $s^r$ and $d^r$ are the source and destination nodes ($s^r, d^r \in V$), and $b^r$ is the traffic demand in qubit(s) of request *r*. Each request *r* has an E2E fidelity threshold $t^r$ to maintain the quality of transmission (QoT). $a^r$ and $\partial ^r$ are the arrival and deadline windows of *r*. The execution time of any request *r* is a window ($h^r$ = 1) and must complete its execution before the deadline $\partial ^r$. In other words, request *r* must be placed in the set of windows $\{a^r+1, a^r+2,...,\partial ^r\}$, otherwise it will be blocked.

The objective of the proposed resource allocation model is to design a distribution model of routing and E2E entanglement so that the number of blocked requests is minimum. In other words, the optimization model aims to place maximum number of requests in each window so that the total number of placed requests is maximum.

### Symmetric purification for bit-flip channel model

In case of a routing path involving multiple intermediate nodes, the E2E fidelity of Bell pairs degrades exponentially with the number of intermediate nodes. If $F_{\text {ini}}$ is the initial fidelity of a Bell pair and *L* is the number of intermediate nodes, then the final fidelity of E2E *F* is expressed using Eq. (1)^14,17^.

1 $$\begin{aligned} F = \frac{1}{4} + \frac{3}{4}(\frac{4F_{\text {ini}}-1}{3})^{L+1} \end{aligned}$$

With NISQ constraints, $F_{\text {ini}}<$1, resulting in exponential degradation of *F* with increasing value of *L*. From Eq. (1), the boundary value of *L* is obtained for a specific value of *F* and it is shown in Eq. (2)^17^. Therefore, Eq. (2) limits the length of a routing path for given values of $F_{\text {ini}}$ and *F*. A potential solution to overcome this limitation is the entanglement purification operation to preserve and improve the fidelity value of the Bell pairs^18–21^.

2 $$\begin{aligned} L+1 \le \lfloor \frac{\text {log} (\frac{4F-1}{3})}{\text {log} (\frac{4F_{\text {ini}}-1}{3})} \rfloor \end{aligned}$$

For example, in case of the bit-flip channel model, an entangled pair $\vert {\Psi ^+} \rangle$ may experience a bit-flip error and results the mixed state shown in Eq. (3) using density operator *ρ*^19,23^, where $\vert {\Phi ^+} \rangle$ denotes a maximally entangled Bell pair..

3 $$\begin{aligned} \rho = F\vert {\Phi ^+} \rangle \langle {\Phi ^+}\vert + (1-F) \vert {\Psi ^+} \rangle \langle {\Psi ^+}\vert \end{aligned}$$

In the entanglement purification operation for the bit-flip channel model^18,20^, two CNOT operations are performed between the qubits from two entangled pairs at either the source or destination node. Next, the second pair of qubits is measured in the computational basis. If the measurement outcomes of the two qubits are identical, the first pair is retained; otherwise, it is discarded. This purification step succeeds when both the entangled pairs either experience a bit-flip error or remain error-free^18,20,22^.

The goal of entanglement purification is to retain the entangled pair with the highest fidelity, sacrificing the other pair and consuming its resources from the network^18,20,22^. If the input pairs have fidelity values $F_1$ and $F_2$, the probability of success for the purification process is $F_1 F_2 + (1-F_1)(1-F_2)$, while the failure probability is $F_1 + F_2 -2F_1 F_2$. The resulting fidelity ($F_{12}$) after a successful purification is given by Eq. (4a)^18,22^.

4a $$\begin{aligned} F_{12} = \frac{F_1 F_2}{F_1 F_2 + (1-F_1)(1-F_2)} \end{aligned}$$

4b $$\begin{aligned} F^{\prime } = \frac{F^2}{F^2 + (1-F)^2} \end{aligned}$$

4c $$\begin{aligned} F^{\prime }_N = \frac{F^2_{N-1}}{F^2_{N-1} + (1-F_{N-1})^2} \end{aligned}$$

From Eq. (4a), the output fidelity $F_{12}$ is greater than $F_1$, $F_2$ only if $F_1, F_2> 0.5$^18,22,23^. If any of the $F_1$ or $F_2$ is less than 0.5, the output fidelity is not beyond the input fidelity values. In case of a symmetric purification model, the Bell pair source is ideal between any adjacent node pair. Therefore, the generated Bell pairs have the same fidelity values between any adjacent node pair. Hence, setting $F_1$ = $F_2$, the final fidelity ($F^{\prime }$) is given by Eq. (4b)^18^. The resultant fidelity after performing the *N*-round of symmetric purification ($F^{\prime }_N$) using mathematical induction is given by Eq. (4c).

## E2E entanglement purification in quantum networks

This section provides a detailed description of the existing LEP model^22,23^ and the proposed PEP model.

### The LEP model

Let request *r* select a routing path from its source node $s^r$ to destination node $d^r$, consisting of a set of nodes ${I_1, I_2, \ldots , I_{l+1}}$, where $s^r = I_1$ and $d^r = I_{l+1}$. Therefore, *l* number of edges in total in that path. The LEP model ensures that the fidelity of the Bell pairs at each of these *l* edges is enough to overcome the degradation of their fidelity due to the entanglement swapping operation^22,23^. Let the fidelity of the Bell pairs on the edge $(I_i, I_{i+1})$ along the routing path of *r* be denoted by $f_i$. To satisfy the E2E fidelity threshold $t^r$, the inequality given in Eq. (5) must hold.

5 $$\begin{aligned} \prod _{i=1}^{l} f_i \ge t^r \end{aligned}$$

If the Bell pairs on each edge involved in the selected path are purified such that they achieve a minimum but sufficient fidelity value of $f^r$ sufficient to meet the fidelity threshold $t^r$, then the constraint described in Eq. (6) must be satisfied.

6 $$\begin{aligned} \prod _{i=1}^{l} f^r \ge t^r \implies (f^r)^{l} \ge t^r \implies f^r \ge (t^r)^{\frac{1}{l}} \end{aligned}$$

Let $F^r_l$ denote the minimum fidelity required for Bell pairs on each edge along the routing path of request *r*, which consists of *l* edges. Then, $F^r_l = (t^r)^{\frac{1}{l}}$, and Eq. (6) can be rewritten as Eq. (7).

7 $$\begin{aligned} f^r \ge F^r_l = (t^r)^{\frac{1}{l}} \end{aligned}$$

**Fig. 2:**
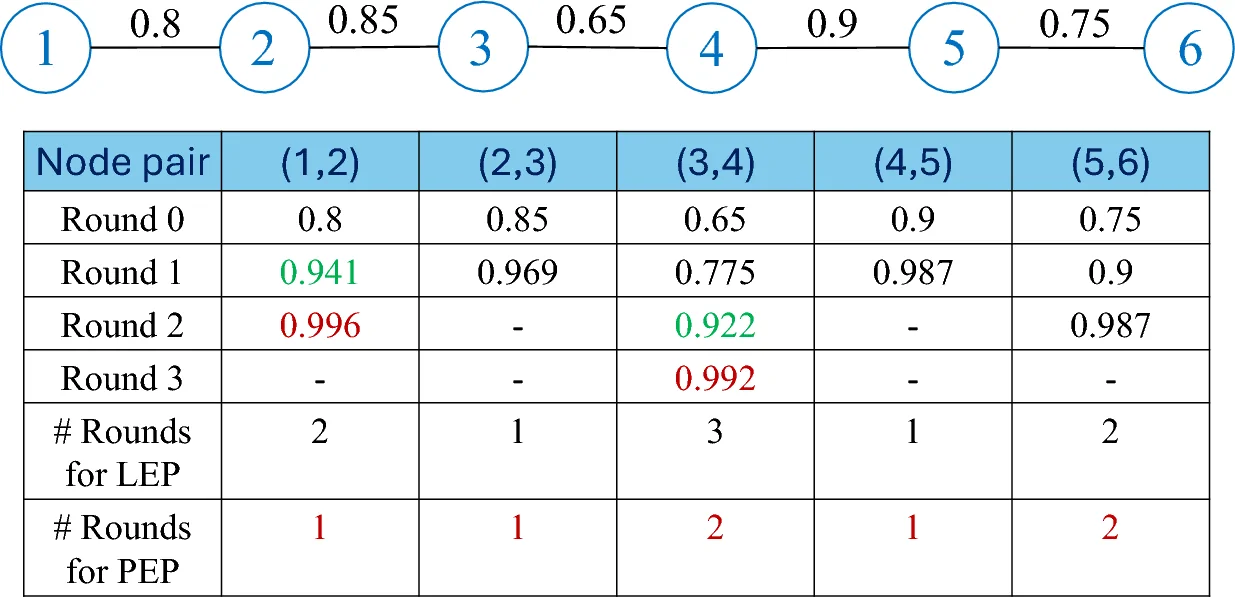
Illustration of the LEP and PEP models in quantum networks.

The concept of the LEP model is illustrated using a sample routing path shown in Fig. 2. In this path, the label in each adjacent node pair represents the fidelity of successfully distribute Bell pairs, e.g., for node pair (1, 2), the fidelity value of Bell pairs is 0.8. A request is considered with source node $s^r$=1, destination node $d^r$=6, demanded traffic $b^r$= 2 quantum channels, and threshold fidelity $t^r$=0.8, respectively. As the number of edges (*l*) in this paths is 5, therefore $F^r_l$=$(t^r)^{\frac{1}{l}}$=$0.8^{\frac{1}{5}}$=0.956. Therefore, the Bell pairs on each of the edges (1, 2), (2, 3), (3, 4), (4, 5), and (5, 6) must be purified until their fidelity value is greater than or equal to $F^r_L$=0.965, as stated in Eq. (7).

As shown in Fig. 2, the number of purification round(s) required in the edges (1,2), (2,3), (3,4), (4,5) and (5,6) to exceed $F^r_L$ (=0.956) is 2, 1, 3, 1, and 2, respectively. The E2E fidelity ($F_K$) for *r* along this path is 0.996*×*0.969*×*0.992*×*0.987*×*0.987= 0.932 which is greater than $t^r$=0.8. As 2 Bell pairs are consumed by each purification operation, required number of Bell pairs for one quantum channel at those edges are $2^2$=4, $2^2=1$=2, $2^3$=8, $2^1$=2, and $2^2$=4, respectively.

### The PEP model

The PEP model works on a specific routing path and considers the results obtained from the LEP model for this purpose. In this model, a set of simple and shortest routing paths is precomputed for each source-destination pair. Let $P^r$ denotes the set of *K* such paths for request *r*, the $k^\textrm{th}$ precomputed path of request *r* is denoted by path (*r*, *k*). The procedure to reduce the number of purification rounds required for path (*r*, *k*) in the PEP model is described below. The PEP model uses Algorithm 1 to compute the possible reduction of purification rounds obtained from the LEP model.

**Algorithm 1:**
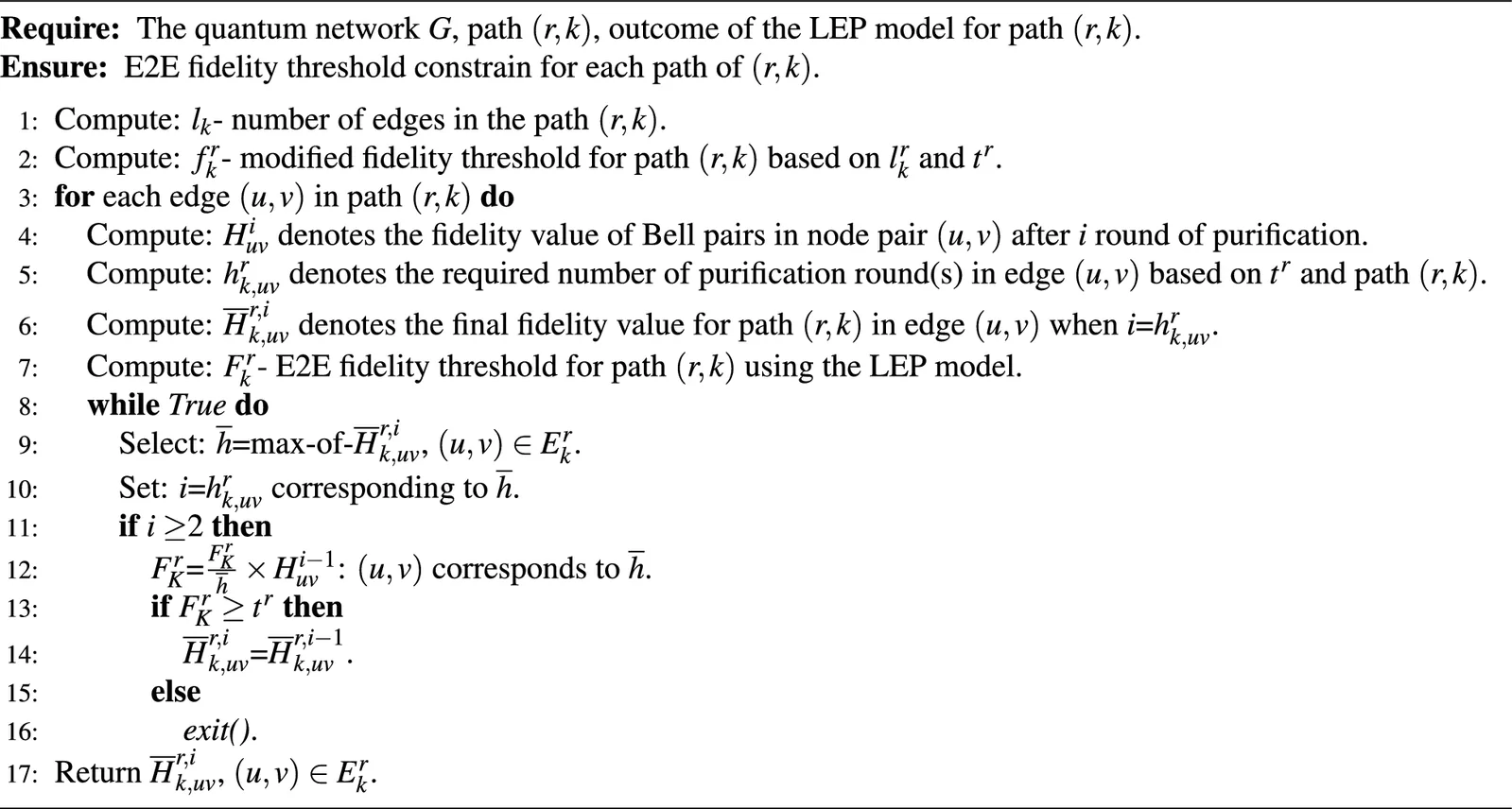
Implementation of the PEP Model for path (*r*, *k*).

The aforementioned idea is illustrated in Fig. 2 with the sample request and the route path aforementioned. In case of node pair (1, 2) in Fig. 2, $H^1_{12}$ = 0.941 and $H^2_{12}$ = 0.996, $h_{k,12}$ = 2 and $\overline{H}^{i}_{k,12}$ = 0.996 for *i* = 2 (=$h_{k,12}$). Similarly, $\overline{H}^{i}_{k,23}$ = 0.969, $\overline{H}^{i}_{k,34}$=0.992, $\overline{H}^{i}_{k,45}$=0.987, and $\overline{H}^{i}_{k,56}$=0.987 for *i*=1, 3, 1, and 2, respectively. As $\overline{H}^{2}_{k,12}$ is maximum (shown in red), *h*=0.996, and $H^{1}_{k,12}$=0.941. Using Steps 2 and 3, $F_K$=$\frac{0.932}{0.996} \times 0.941$= 0.879, which is greater than $t^r$=0.8. *Hence, one less purification round is required in between node pair (1, 2) using the PEP model.* In this way, steps 2 and 3 are repeated for node pair (3, 4) and $F_K$=$\frac{0.879}{0.992} \times 0.922$= 0.816. *One less purification round is required in between node pair (3, 4).* It can be noted that $F_k$ cannot be reduced further for $t^r=0.8$. The number of purification rounds (s) required in edges (1,2), (2,3), (3,4), (4,5) and (5,6) are 1, 1, 2, 1 and 2, respectively; two rounds less than those of the LEP model. As 2 pairs of Bell are consumed by each purification operation, the number of pairs of Bell required for one quantum channel at these edges is $2^1$ = 2, $2^2=1$ = 2, $2^2$ = 4, $2^1$ = 2 and $2^2$ = 4, respectively.

### Analysis of performance of the LEP and PEP models

It is evident that the reduction of the number of purification rounds (s) by the PEP model compared to the LEP model depends on the (i) fidelity threshold $t^r$, and (ii) fidelity of the Bell pairs in the link(s) of the routing path. As the PEP model targets the deviation of $t^r$ and the achieved E2E fidelity ($F_K$), a higher value of $t^r$ results the similar results from the LEP and PEP model. On the other hand, if the fidelity values of Bell pairs in the links of a path is low, more rounds of purification are required in those links by the LEP model, which allows the PEP model for better optimization.

In case of the LEP model, the entanglement purification operations are performed in the link level and on the Bell pairs stored in the quantum memories. In case of the PEP model, the entanglement purification operations are also performed in the link level; same as the LEP model. This is due to the fact that, for the PEP model, one or multiple purification operation(s) are less performed (if required) in any link. Hence, similar to the LEP model, the purification operations are performed on the Bell pairs stored in the memories in the nodes.

An entanglement purification operation for bit-flip channel model includes two local CNOT gate operations at the node pairs, a measurement operation, and transmit the measurement results^19^. The time required for performing this purification operation is on the order of milliseconds (ms)^33^. If multiple purification operations are required, they are performed sequentially between any node pair. On the other hand, the decoherence times of quantum memories are on the order of seconds (s)^5,34^. Therefore, even if the input fidelity is too low (e.g., $F_1, F_2 \approx 0.51$), the LEP and PEP models are able to perform multiple purification operations on the Bell pairs stored in the memories so that the fidelity value is increased beyond the fidelity threshold $t^r$.

## Proposed resource allocation model

The E2E fidelity-aware resource allocation model is divided into following steps: (i) computation of required Bell pairs to ensure E2E fidelity, (ii) allocation of required Bell pairs in the selected path for each request in the network.

### Step 1: Computation of required Bell pairs to ensure E2E fidelity

A set of precomputed simple shortest paths is computed for each request *r ∈ R* using Yen’s Algorithm^35^. Let $P^r$ be the set of such paths for request *r ∈ R* in sorted order of their length in number of hops. The path $k^\textrm{th}$ ($k \in |P^r|$) of the request *r* is denoted by path (*r*, *k*). The required number of Bell pairs is calculated to ensure the E2E fidelity-aware entanglement distribution at each edge for each path (*r*, *k*) as follows.

(i) **Perform purification using the LEP (PEP) model:** Let $\overline{x}^{rk}_{uv}$ ($x^{rk}_{uv}$) denote the number of purification rounds on the edge (*u*, *v*) for the path (*r*, *k*) based on $t^r$ and $F_{uv}$ using the LEP (PEP) model. The required number of Bell pair(s) in edge (*u*, *v*) for E2E fidelity-aware routing of 1 entanglement channel is $2^{\overline{x}^{k}_{uv}}$ ($2^{x^{k}_{uv}}$) for the LEP (PEP) model. Let $y^{k}_{uv}$= $2^{\overline{x}^{k}_{uv}}$ ($2^{x^{k}_{uv}}$) for the LEP (PEP) model.

(ii) **Incorporation of ESP:** Let $I^{rk}_\textrm{int}$ be the set of intermediate nodes (s) on the path (*r*, *k*). Let $Q^{rk}_\textrm{int}$ be the product of the ESP(s) of the node(s) from the set $I^{rk}_\textrm{int}$, that is, $Q^{rk}_\textrm{int} =\prod _{u \in I^{rk}_\textrm{int}} Q_u$. The number of Bell pairs required to establish the $b^r$ number of entanglement channels between nodes $s^r$ and $d^r$ along the (*r*, *k*) path is $z^{rk}$ = $\lceil \frac{b^r}{Q^{rk}_\textrm{int}} \rceil$.

(iii) **Final Computation:** The number of Bell pairs required in edges (*u*, *v*) along the selected path of *r* considering the fidelity threshold $t^r$ and the ESP is $\gamma ^{rk}_{uv}$= $y^{rk}_{uv} z^{rk}$.

The parameter $\gamma ^{rk}_{uv}$ is computed and provided as input to the next step.

**Illustration:** To illustrate these steps, we consider the routing path mentioned in Fig. 2. Let the ESP of each node in the set $I^{rk}_\textrm{int}$ = $\{2,3,4,5\}$ be 0.9, then $Q^{rk}_\textrm{int} = 0.9^4 = 0.6561$. Let $b^r$ = 3, then $z^{rk}$=$\lceil \frac{3}{0.6561}\rceil$ = 5.

*∙* **For the LEP model**, the number of purification rounds required in the edges (1,2), (2,3), (3,4), (4,5) and (5,6) using the LEP model ($\overline{x}^{rk}_{uv}$) are 2, 1, 3, 1, and 2, respectively. Hence, the values of $y^{k}_{uv}$ = $2^{\overline{x}^{rk}_{uv}}$ for those edges are 4, 2, 8, 2 and 4, respectively. As the value of $z^{rk}$ is 5, the value of $\gamma ^{rk}_{uv}$ for the edges (1,2), (2,3), (3,4), (4,5), and (5,6) are 20, 10, 40, 10, and 20, respectively.

*∙* **For the PEP model**, the number of purification rounds required in the edges (1,2), (2,3), (3,4), (4,5) and (5,6) using the LEP model ($x^{rk}_{uv}$) are 1, 1, 2, 1, and 2, respectively. Hence, the values of $y^{k}_{uv}$ = $2^{\overline{x}^{rk}_{uv}}$ for those edges are 2, 2, 4, 2 and 4, respectively. As the value of $z^{rk}$ is 5, the value of $\gamma ^{rk}_{uv}$ for the edges (1,2), (2,3), (3,4), (4,5) and (5,6) are 10, 10, 20, 10, and 20, respectively.

From the aforementioned illustration, it is observed that the PEP model requires 10 and 20 Bell pairs less compared to the LEP model in the edges (1,2) and (3,4) in identical setup.

### Step 2: Resource allocation model

The online quantum network with time-slotted quantum operation is considered where *W* is the set of windows, and each window has a cutoff decoherence time of quantum memories $t_c$. At the beginning of each window *w ∈ W*, *M* is the number of Bell pairs distributed in each edge (*u*, *v*) in the network with probability $P_{uv}$. Let $M^{w}_{uv}$ denote the number of successfully distributed Bell pairs on edge (*u*, *v*) on window *w*. As the symmetric purification model is considered, all Bell pairs distributed between each node pair (*u*, *v*) have the same fidelity.

The resource allocation model is designed considering two approaches-ILP-based formulation and greedy heuristic-based algorithm. They are described as follows.

#### ILP formulation-based Heuristic

The ILP-based heuristic is described in Algorithm 2, which executes the ILP for each window *w ∈ W* to place requests in the network. Based on the arrival time $a^r$ and the deadline $\partial ^r$ of the request *r*, the set of windows eligible to be placed *r* is calculated as: $W^r= \{a^r+1, a^r+2,...,\partial ^r\}$.

The Algorithm 2 works as follows. In each window, first the Bell pairs are distributed in the network. Next, the number of successfully distributed Bell pairs is computed for each node pair. Then the set of unallocated requests to be placed in the window *w* ($R_w$) is computed. Next, the procedure Place-ILP $(R_w)$ is called which aims to place request set $R_w$ in the network with capacity set $M^w_{uv}$. The objective of this procedure Place-ILP $(R_w)$ is to place the maximum number of requests from the set $R_w$ in the network constrained by the set of capacity *M*. The procedure Place-ILP formulation is described in Eq. (8)-Eq. (13).

**Algorithm 2:**
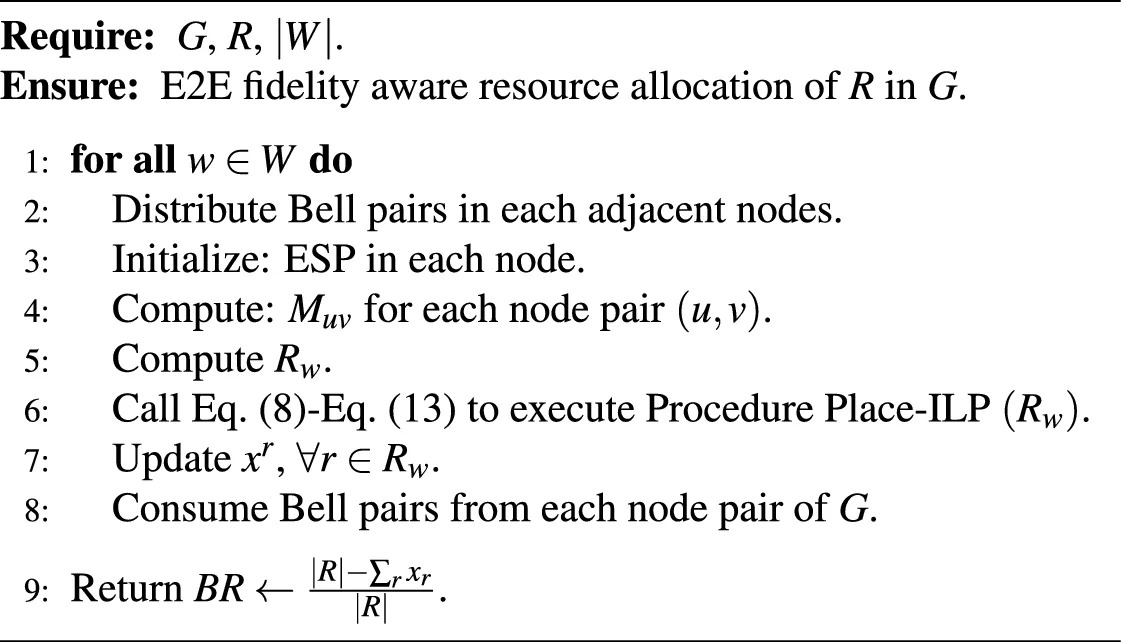
ILP formulation-based Heuristic Algorithm.

The set of constants and parameters used in this ILP formulation are described as follows. $R_w$: The set of unallocated requests to be placed in the window *w*. $A^r_{k,uv}$: An input binary constant, 1 if edge (u v) is in the path (*r*, *k*), and 0, otherwise. $\gamma ^r_{k,uv}$: An integer constant denoting the number of Bell pairs required for purification in the edge (u v) of the path (*r*, *k*) using LEP (PEP) model. $M_{uv}$: An integer constant denoting the number of Bell pairs successfully distributed between node pair (*u*, *v*).

The variables used in this ILP formulation are described as follows.

$x^r$: A binary variable, 1 if request *r* is placed. and 0, otherwise.

$\alpha ^r_k$: A binary variable, 1 if the path (*r*, *k*) is selected for request *r*, and 0, otherwise.

$\beta ^r_{k,uv}$: A binary variable, 1 if edge *(u\quad v)* is used by the path (*r*, *k*) and $\alpha ^r_k$=1, and 0, otherwise.

$\delta ^r_{k,uv}$: A non-negative integer variable denoting the number of Bell pairs required for purification in the edge *(u\quad v)* of the path (*r*, *k*).

$\gamma _{uv}$: A non-negative integer variable denoting the total number of Bell pairs required in the edge *(u\quad v)*.

The ILP formulation is described below.

8 $$\begin{aligned} \text {Minimize:} \quad |R_w|-\sum _{r \in R_w} x^r \end{aligned}$$

Subject to the following constraints.

9 $$\begin{aligned} \sum _{k \in K} \alpha ^r_k = x^r, \quad \forall r \in R_w \end{aligned}$$

10 $$\begin{aligned} \beta ^r_{k,uv} = A^r_{k,uv} \alpha ^r_k, \quad \forall r \in R_w, k, (u,v) \end{aligned}$$

11 $$\begin{aligned} \delta ^r_{k,uv} = \beta ^r_{k,uv} \gamma ^r_{k,uv}, \quad \forall r \in R_w, k, (u,v) \end{aligned}$$

12 $$\begin{aligned} \gamma _{uv} = \sum _{r \in R_w}\sum _{k \in K} \delta ^r_{k,uv}, \quad \forall (u,v) \end{aligned}$$

13 $$\begin{aligned} \gamma _{uv} + \gamma _{vu} \le C_{uv}, \quad \forall (u,v) \end{aligned}$$

Eq. (8) states the objective of this ILP formulation- minimize the number of unallocated requests in the network. Eq. (9) states that the value of $x^r$ is set to 1 if any path from the set $P^r$ is selected. Eq. (10) states that if path (*r*, *k*) is selected ($\alpha ^r_k$=1), the edge(s) included in path (*r*, *k*) is (are) marked as selected by setting $\beta ^r_{k,uv}$=1. If edge *(u → v)* is selected by path (*r*, *k*), Eq. (11) computes the required number of Bell pairs including ESP and purification for $b^r$ on that edge. Eq. (12) computes the total number of Bell pairs utilized in the node pair (*u*, *v*) in the direction *(u → v)*. Eq. (13) ensures that the total Bell pair is required in the node pair (*u*, *v*) in both directions *(u → v)* and *(v → u)* must be less than the successfully distributed Bell pairs in that node pair ($M_{uv}$).

The number of binary variables in this ILP formulation is $\mathcal {O}(|R||K| + |R||K||V|^2) \approx \mathcal {O}(|R||K||V|^2)$. The number of integer variables in this ILP formulation is $\mathcal {O}(|R||K||V|^2)$. This ILP formulation is executed in each window *w ∈ W*, and the number of constraints in this ILP formulation is $\mathcal {O}(|R||K||V|^2)$.

#### Greedy approach-based heuristic

The greedy approach-based heuristic for resource allocation is described in Algorithm 3, which calls the procedure Place-Heu($R_w$) for each window *w ∈ W* to place the request set $R_w$ in the network. After computing $\gamma ^{rk}_{uv}$ for each path (*r*, *k*), requests are placed with the objective of minimizing the blocking ratio (*BR*).

For resource allocation in each window, an increasing relative probability value $\rho ^r$ is computed for placing request *r* in any window *w*, based on $|W^r|$. The computed values of $\rho ^r$ ensure that the request *r* has a lower chance of being placed in *G* during the earlier windows, and this probability increases as the index of window $w \in W^r$ increases. Consequently, for the last window $w \in W^r$, the value of $\rho ^r$ is always equal to 1. Initially, all requests in the set *R* are marked as unplaced. Let $x^r$ denote the placement status of request *r*, initialized as $x^r \leftarrow 0$. Here, $R_w$ represents the set of unallocated requests to be placed in window *w*. Within each window *w*, the requests in $R_w$ are sorted in increasing order of $\partial ^r - w$; in other words, the requests with earlier deadlines are processed first.

**Algorithm 3:**
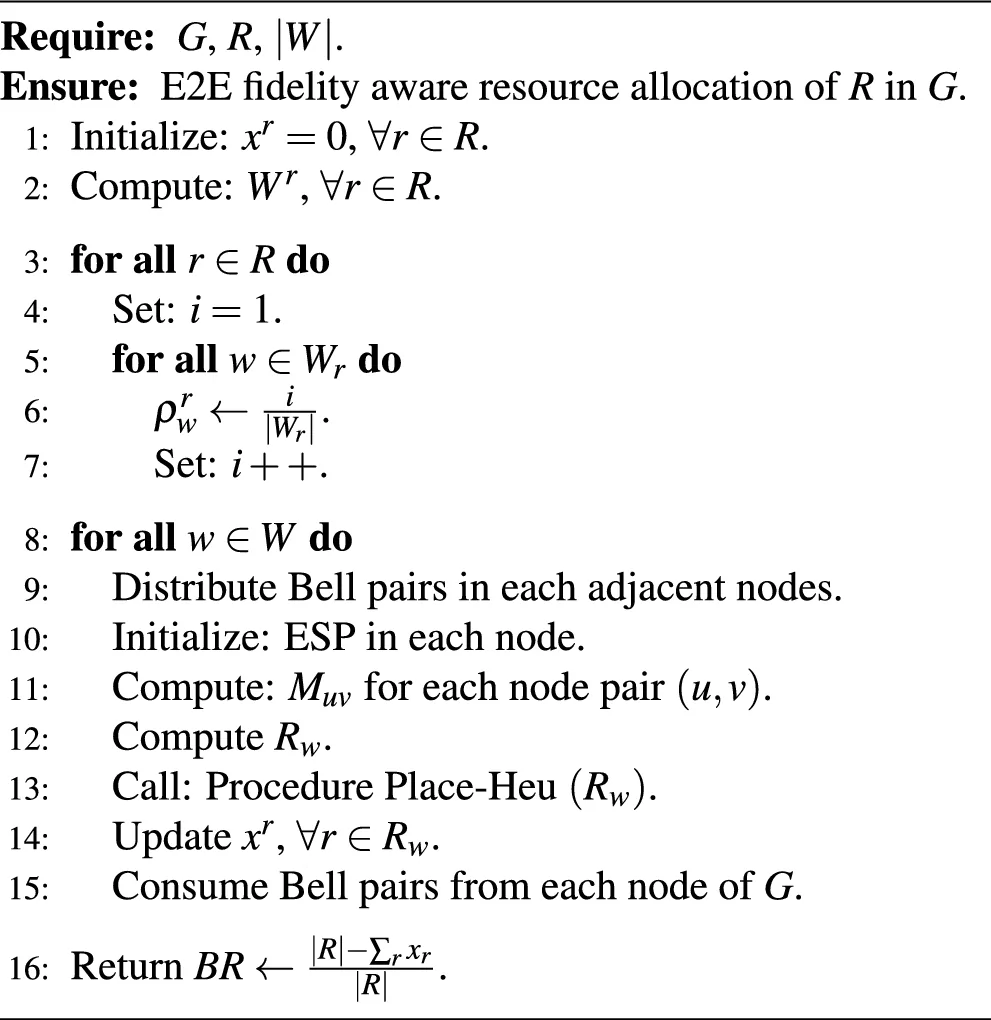
Greedy approach-based Heuristic Algorithm.

The procedure Place-Heu is described in Algorithm 4 which operates for each window *w ∈ W*. For each request, it searches for a suitable path that is (i) shorter in length (in terms of the number of hops) and (ii) has a higher value of $M_{uv}$. For each request *r* and path (*r*, *k*), the bottleneck capacity $BC^r_k$–defined as the minimum number of Bell pairs between any pair of nodes along the path–is calculated. Let $\phi ^r_k$ denote the maximum value of $\gamma ^{rk}_{uv}$ for all node pair (*u*, *v*) on the path (*r*, *k*). If $BC^r_k \ge \phi ^r_k$, i.e., there are sufficient resources along that path, then path (*r*, *k*) is considered eligible to place the request *r* in window *w*. Let $l^r_k$ be the number of edges in the path (*r*, *k*). A path with a smaller value of $l^r_k$ is more efficient for the allocation of resources to the request *r*. Hence, a higher value of $\frac{1}{l^r_k}$ indicates a more desirable path. Set: $\overline{\phi }^r_k = BC^r_k - \phi ^r_k$; a higher value of $\overline{\phi }^r_k$ also indicates a more favorable path for resource allocation of *r*. Now, let $\psi ^r_k = \frac{\overline{\phi }^r_k}{l^r_k}$. Based on the above criteria, a higher value of $\psi ^r_k$ represents a better path for the placement of request *r*. The Place-Heu procedure therefore selects the path with the maximum $\psi ^r_k$ to place *r* in the network *G* in window *w*. If request *r* is successfully placed in *G* within this window, then $x^r$ is set to 1.

The computational complexity of Algorithm 3 is determined as follows. The computational complexity of *K*-shortest path using Yen’s Algorithm^35^ for the request set *R* is $\mathcal {O}(|R|K(|E|+|V|log|V|))$. The worst-case complexity of lines 3–7 of Algorithm 3 is $\mathcal {O}(|R||W|)$. For each window *w ∈ W*, Algorithm 3 calls procedure Place-Heu to place each request $r \in R_w$. The computational complexity of procedure Place-Heu is $\mathcal {O}(|R|log|R| + |R|K|V|^2)$. Hence computational complexity of Algorithm 3 is $\mathcal {O}(|R|K(|E|+|V|log|V|) + |R||W| + |W|(|R|log|R| + |R|K|V|^2))$.

**Algorithm 4:**
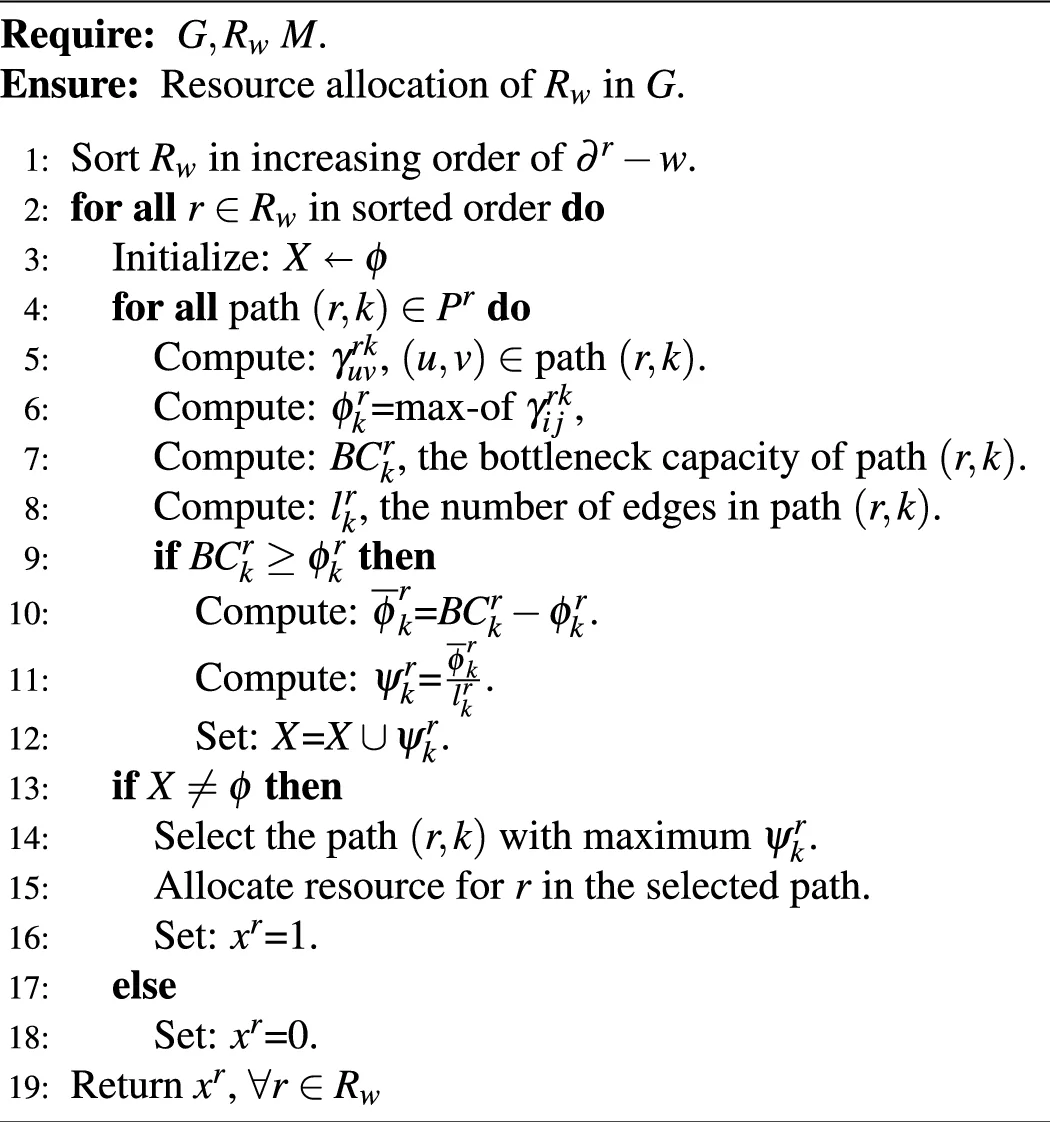
Procedure Place-Heu.

## Experimental results

This section provides a performance comparison of the developed ILP formulations and heuristic algorithms of the LEP and PEP models in terms of (i) the blocking ratio (*BR*), (ii) the fidelity of E2E ($$F_{E2E})$$, and (iii) the computational time (*CT*) [in seconds]. First, the performance of the ILP formulation of the LEP and PEP models is compared, then the performance of the corresponding greedy heuristics is evaluated.

### Simulation results from ILP formulations

*∙* **Simulation Setup:** The number of requests is varied from 10 to 30, with a step of 5. The traffic demanded $b^r$ is uniformly distributed in the range [2, 6]. The threshold fidelity $t^r$ is selected from the set $\{0.8, 0.85, 0.9\}$. The value of EDP for each adjacent node pair and the value of ESP for each node are set to 0.8, respectively. The fidelity values of the distributed Bell pairs between adjacent node pairs are uniformly distributed from the set $\{0.65, 0.7, 0.75, 0.8, 0.85, 0.9, 0.95\}$. The number of Bell pair generation windows |*W*| is set to 4. The value of *M* is varied from 12 to 20, with a step of 4. Two 3*×*3 and 4*×*4 square-grid quantum networks are considered to evaluate the performance of the ILP formulations. The number of precomputed paths (*K*) is set to 2 and they are computed using Yen’s Algorithm^35^. The simulation is performed 20 times for each parameter considered and the result is obtained with a confidence interval of 95%. The arrival time $a^r$ of the request set is generated by the Poisson distribution with parameters $(\frac{|W|}{4},|R|)$, and the deadline of each request is randomly selected from the range $[a^r +1, |W|]$^10^. The ILP formulations are evaluated using the IBM ILOG CPLEX optimization studio (v12.8)^36^.

**Fig. 3:**
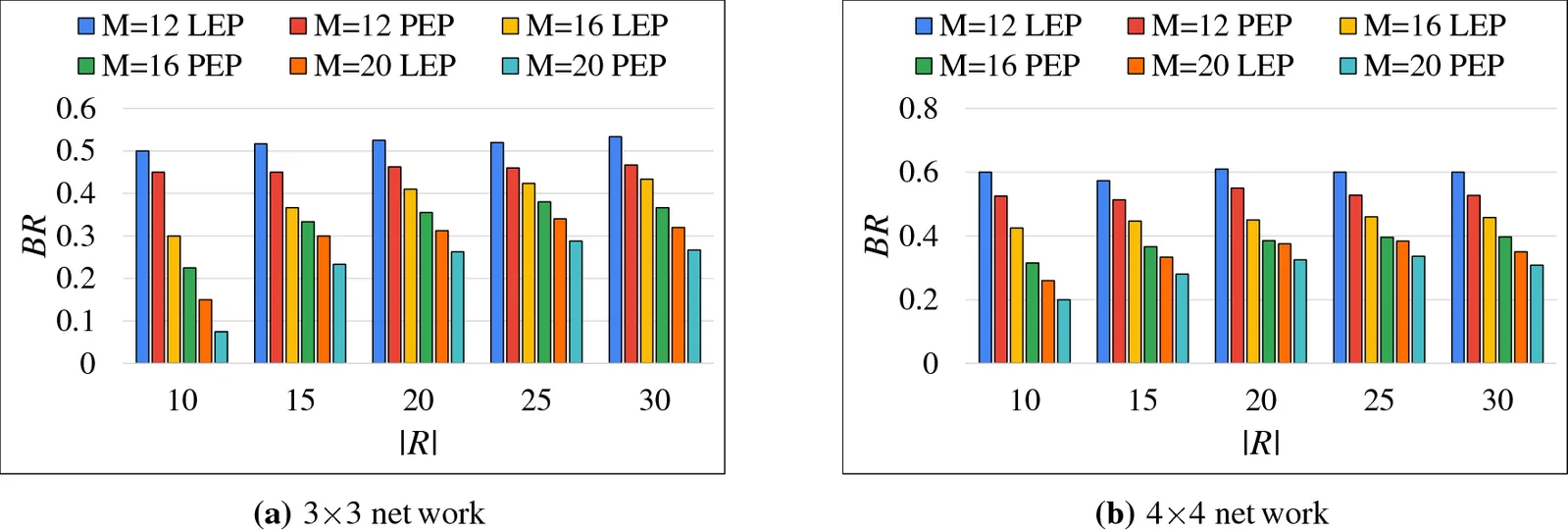
Variation of *BR* with |*R*| and |*M*| for ILP-based approach.

**Table 1:** Variation of E2E fidelity ($F_{E2E}$) and *CT* [in seconds] with threshold fidelity *t* and network size for the LEP and PEP models.

$t^r$	Model	$F_{E2E}$		CT [Sec]	
		3x3	4x4	3x3	4x4
0.8	LEP	0.895	0.876	1457	2174
	PEP	0.843	0.833	1374	2153
0.85	LEP	0.883	0.873	1683	2449
	PEP	0.862	0.857	1649	2427
0.9	LEP	0.937	0.925	1854	2773
	PEP	0.918	0.913	1836	2719

*∙* **Results Obtained:** The variation of *BR* with the number of requests |*R*| and the number of memories between the pairs of adjacent nodes *M* is reported in Fig. 3. The following observations are concluded from the Figure 3. The value of *BR* decreases for both the LEP and PEP models when *M* increases, as expected. For example, in the 3*×*3 network (Fig. 3a), *BR* decreases from (i) 0.525 to 0.41 for the LEP model (a decrease of 21.9%) and (ii) 0.4625 to 0.355 for the PEP model (a decrease of 27.56%), when the value of *M* increases from 12 to 16 for |*R*| = 20. As the PEP model reduces the number of purification rounds, and hence the required number of Bell pairs per request, the PEP model outperforms the LEP model in terms of *BR* in all cases. For example, in the 3*×*3 network (Fig. 3a), *BR* decreases from 0.41 to 0.355 (a decrease of 13.41%) for the PEP model compared to the LEP model when *M*=16 and |*R*| = 20.

Table 1 reports the variation of E2E fidelity ($F_{E2E}$) and the computational time (*CT*) [in seconds] with the fidelity threshold *t* and network size for the ILP formulations for the LEP and PEP models considering *B ∈ [2, 6]*. The following observations are summarized from the obtained results. The values of $F_{E2E}$ are higher for the LEP model compared to the PEP model in the identical simulation setup. Hence, the PEP model compromises the quality of service in terms of E2E fidelity (still maintaining the fidelity threshold) for better resource utilization. For a fixed threshold fidelity $t^r$, the values of $F_{E2E}$ decrease with increasing network size. This is due to the increase in the number of intermediate nodes when the size of the network increases, which results in lower values of $F_{E2E}$. The computation time (*CT*) increases with increasing network size, and it is comparable for the LEP and PEP models in an identical simulation setup.

### Simulation results from heuristic

**Fig. 4:**
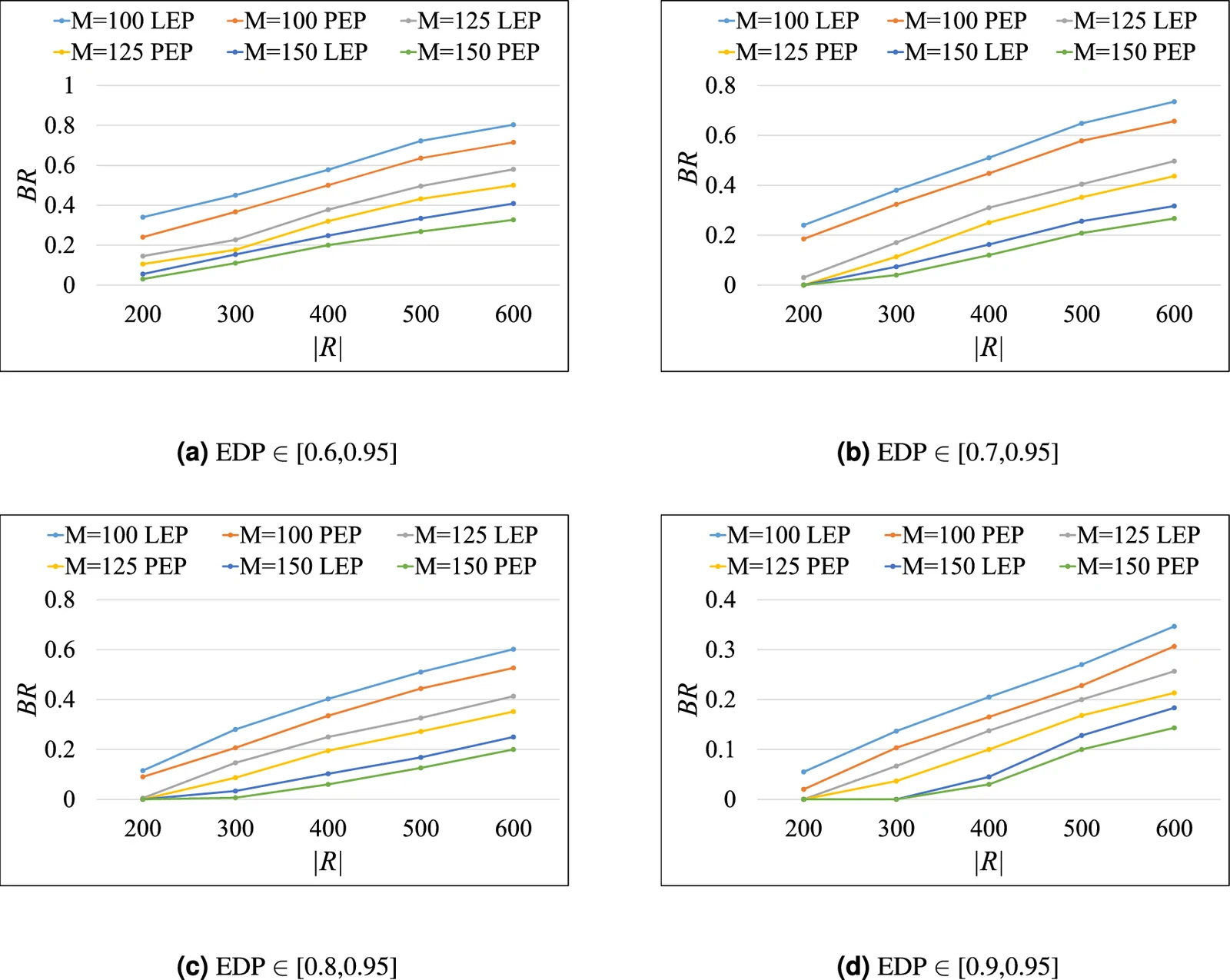
Variation of *BR* with |*R*| and |*M*| for varying EDP range for heuristic-based approach.

**Fig. 5:**
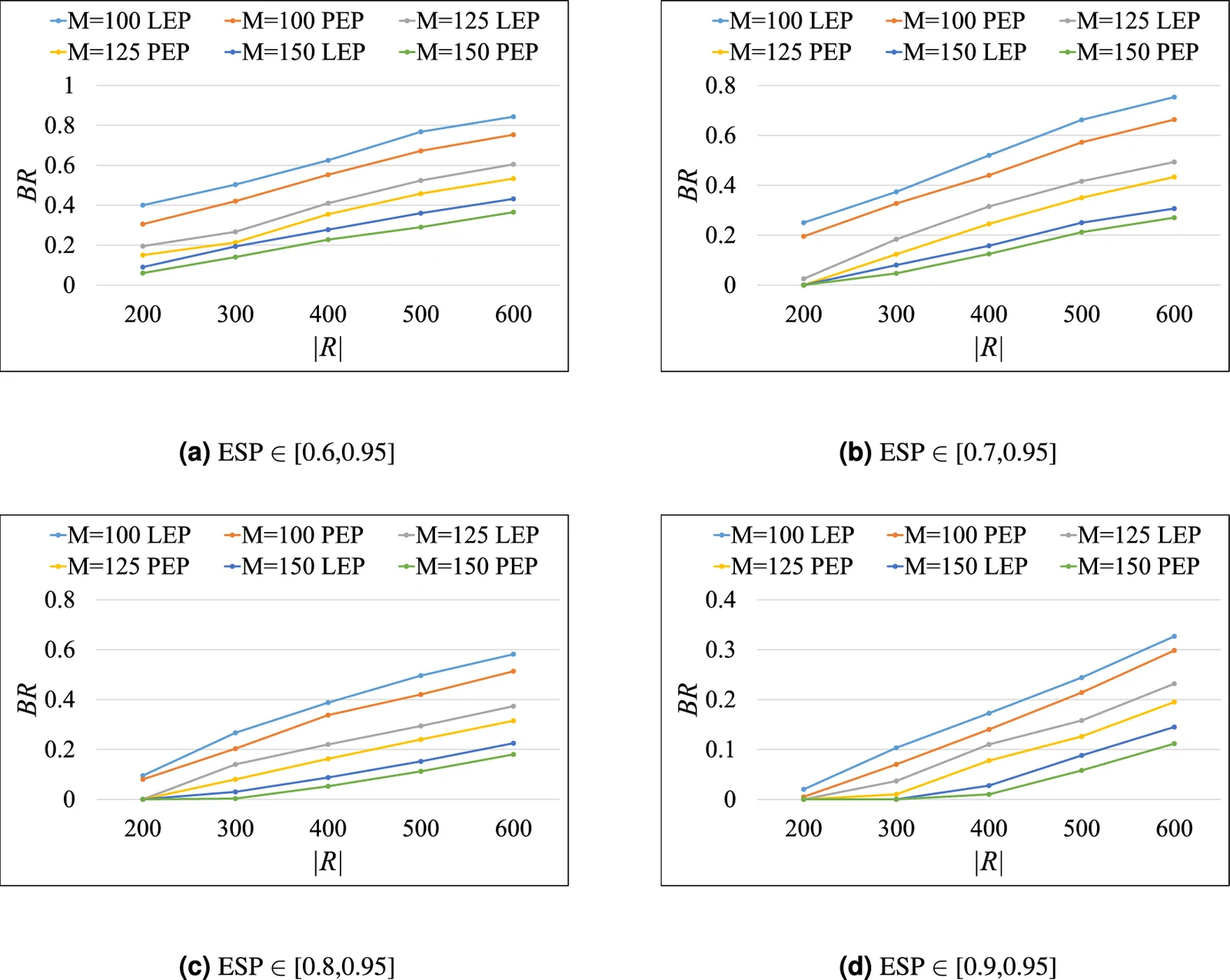
Variation of *BR* with |*R*| and |*M*| for varying ESP range for heuristic-based approach.

- **Simulation Setup:** The number of requests varies from 200 to 600, with a step of 100. The traffic demanded $B^r$ is uniformly distributed in the range [2, 10]. The threshold fidelity $t^r$ is randomly selected from the set $\{0.8, 0.85, 0.9\}$. The fidelity values of the distributed Bell pairs are uniformly distributed between the adjacent node pairs of the set $\{0.65, 0.7, 0.75, 0.8, 0.85, 0.9, 0.95\}$. The number of Bell pair generation windows |*W*| is set to 10. A 5*×*5 mesh quantum network is considered to evaluate the simulation performance. The value of *M* ranges from 100 to 150, with a step of 25. The number of precomputed paths (*K*) is set to 3 and they are computed using Yen’s Algorithm^35^. The simulation is performed 50 times for each parameter considered and the result is obtained with a confidence interval of 95%. The arrival time $a^r$ of the request set is generated by the Poisson distribution with parameters $(\frac{|W|}{4},|R|)$, and the deadline of each request is randomly selected from the range $[a^r +1, |W|]$^10^. Initially, the variation of *BR* is calculated by setting ESP=0.8 for each node and randomly distributing the EDP of each pair of nodes from the range $[P_d,0.95]$, where $P_d \in \{0.6, 0.7, 0.8, 0.9\}$. Next, the variation of *BR* is calculated by setting the EDP=0.8 of each node pair, and randomly distributing the ESP of each node in the range $[P_s,0.95]$, where $P_s \in \{0.6, 0.7, 0.8, 0.9\}$.
- **Results Obtained:** Figures 4 and 5 plot the variation of *BR* with |*R*| and *M* for different ranges of EDP and ESP, respectively. The following observations are summarized from the results obtained from Figs. 4 and 5.

(i) For a given simulation setup, the value of *BR* increases with an increase in the number of incoming requests |*R*|. For example, for EDP *∈ [0.6,0.95]* and *M*=100 (shown in Fig. 4a), values of *BR* increases from 0.34 (0.241) to 0.803 (0.715) (a increase of 136.17% (196.68%)) for the LEP (PEP) model when |*R*| is increased from 200 to 600, respectively.
(ii) For a given simulation setup, the value of *BR* decreases with an increase in the number of Bell pairs distributed among the adjacent node pairs, *M*. For example, for EDP *∈ [0.8,0.95]* and |*R*|=400 (shown in Fig. 4c), values of *BR* are 0.402, 0.25, and 0.1025 (0.335, 0.195, and 0.06) for the LEP (PEP) model when *M* is set to 100, 125, and 150, respectively.
(iii) The PEP model outperforms the LEP model in terms of *BR* in all cases. For example, from Fig. 4bwhen |*R*|=400 and *M*=125, the value of *BR* for the LEP and PEP models are 0.314 and 0.251 (a decrease of 20.06%), respectively. Similarly, from Fig. 5bwhen |*R*|=400 and *M*=125, the value of *BR* for the LEP and PEP models are 0.315 and 0.246 (a decrease of 21.9%), respectively.
(iv) In Figs. 4aand 5a, when both EDP and ESP are in the range [0.6,0.95], the value of *BR* is lower for Figs. 4a, when other parameters are fixed. For example, when |*R*|=400 and *M*=125, the values of *BR* are 0.3755 and 0.32 (a decrease of 14.78%) for the LEP and PEP models when EDP *∈ [0.6,0.95]* (Fig. 4a), and the values of *BR* are 0.41 and 0.355 (a decrease of 13.41%) for the LEP and PEP models when ESP *∈ [0.6,0.95]* (Fig. 5a), respectively. This is because the EDP operates on each edge along the paths and increases the required number of Bell pairs for each quantum channel. However, the ESP operates on $b^r$ and along the E2E entanglement distribution. Hence, for the lower range of EDP and ESP, ESP has the stronger effect on *BR* compared to EDP.
(v) In Figs. 4dand 5d, when both EDP and ESP are in the range [0.9,0.95], the value of *BR* is higher for Figs. 4d, when other parameters are fixed. For example, when |*R*| = 400 and *M* = 125, the values of *BR* are 0.132 and 0.11 (a decrease of 16.67%) for the LEP and PEP models when EDP *∈ [0.9,0.95]* (Fig. 4d), and the values of *BR* are 0.112 and 0.077 (a decrease of 31.25%) for the LEP and PEP models when ESP *∈ [0.9,0.95]* (Fig. 5d), respectively. This is due to the higher range of ESP (near to 1) which has a lower effect on *BR* compared to EDP, which operates on each edge, as mentioned earlier.
(vi) In the case of EDP and ESP ranges [0.7,0.95] and [0.8,0.95], the values of *BR* are comparable for the LEP (PEP) model, when other parameters are fixed.

**Fig. 6:**
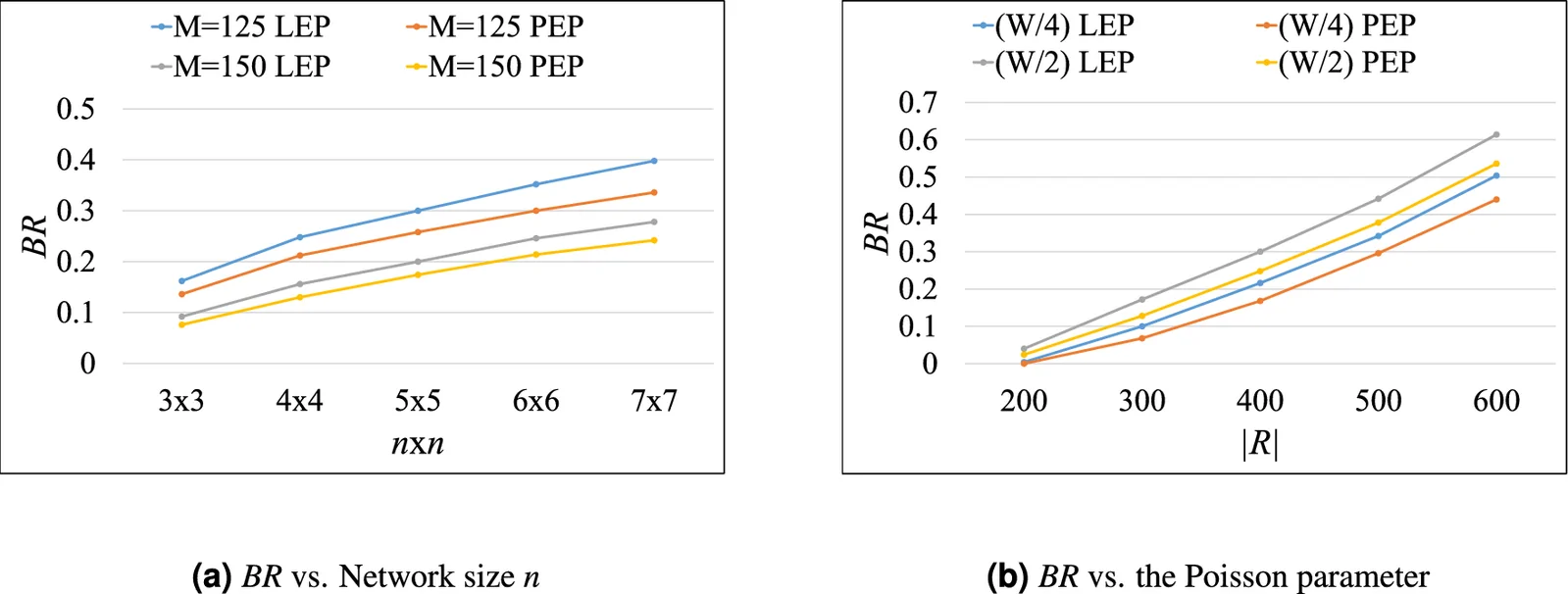
Variation of *BR* with the network size and Poisson parameter used for the traffic model.

**Table 2:** Variation of E2E fidelity ($F_{E2E}$) and *CT* [in seconds] with network size for the LEP and PEP models when threshold fidelity $t^r$ = 0.8.

Parameter	Model	3x3	4x4	5x5	6x6	7x7
CT	LEP	2.3	18.2	128	453	1047
	PEP	2.3	18	128.1	458.5	1043
$F_{E2E}$	LEP	0.895	0.887	0.873	0.865	0.851
	PEP	0.841	0.835	0.827	0.822	0.819

Figure 6 reports the performance of the proposed heuristic for the LEP and PEP models in terms of the variation in network size and the Poisson parameter used to generate online traffic. The number of requests and windows are set to 500 and 10, respectively. The values of EDP and ESP across the network are set to 0.8. The threshold fidelity $t^r$ is randomly selected from the set $\{0.8, 0.85, 0.9\}$. The fidelity values of the distributed Bell pairs are uniformly distributed between the adjacent node pairs of the set $\{0.65, 0.7, 0.75, 0.8, 0.85, 0.9, 0.95\}$. The traffic demanded $B^r$ is uniformly distributed in the range [2, 10]. The value of *M* is set to 125.

Figure 6areports the variation of *BR* with increasing size of the square-grid topology *n× n* where the value of *n* increases from 3 to 7. The value of *M* is set to 125 and 150. It is observed that the value of *BR* increases with increasing value of *n*. For example, the values of *BR* are 0.162 and 0.398 for the LEP model when *n* is set to 3 and 7, respectively, for *M* = 125. The value of *BR* decreases with increasing value of *M* for all values of *n*, as described above. In all cases, the PEP model dominates the LEP model in terms of *BR*.

In Figure 6b, two types of traffic models are considered: (i) the arrival time $a^r$ of the request set is generated by the Poisson distribution with parameters $(\frac{|W|}{4},|R|)$, and (ii) the arrival time $a^r$ of the request set is generated by the Poisson distribution with parameters $(\frac{|W|}{2},|R|)$. In both cases, the deadline for each request is randomly selected from the range $[a^r +1, |W|]$. The number of requests varies from 200 to 600, with a step of 100. The value of *M* is set to 125. In the former case, the deviation of $a^r$ and $\partial ^r$ is higher than in the latter case. In other words, when the Poisson parameter is changed from $\frac{|W|}{4}$ to $\frac{|W|}{2}$, a request has a lower number of eligible window(s) to be placed on the network. Hence, for the Poisson parameter $\frac{|W|}{4}$, the arrival times are well-distributed in the windows [1, |*W*|], while for $\frac{|W|}{4}$, the arrival times are centered on $\frac{|W|}{2}$. Hence, in the latter case, a congestion traffic model is generated in the middle window index, which results in a lower value of *BR* compared to the former one. For example, the values of *BR* are 0.0.342 and 0.441 for the LEP model for the Poisson parameters $\frac{|W|}{4}$ and $\frac{|W|}{2}$, respectively, when |*R*| is set to 500. As expected, the PEP model dominates the LEP model in terms of *BR*.

Table 2 reports the variation of $F_{E2E}$ and *CT* [in seconds] with the network size for the LEP and PEP models when $t^r$ = 0.8. The rest simulation setup is identical with the Fig. 6a. Similarly to the ILP formulation, the values of $F_{E2E}$ are higher for the LEP model compared to the PEP model, and the values of $F_{E2E}$ decrease with increasing network size. Again, the value of *CT* increases with increasing network size and is comparable for the LEP and PEP models in an identical simulation setup.

## Conclusion

In this study, we address the problem of online routing, end-to-end (E2E) entanglement distribution, and purification in quantum networks, and propose an online routing algorithm that ensures fidelity-aware E2E entanglement distribution. In this regard, we extend the existing link-based entanglement purification (LEP) model to a path-based entanglement purification (PEP) model. Both the LEP and PEP models integrate the E2E entanglement distribution with E2E entanglement purification along the routing paths. The proposed heuristic algorithm considers integer linear programming (ILP) and a greedy-based heuristic for optimal path selection and Bell pair allocation. Extensive simulation experiments are conducted to evaluate the performance of the proposed models by varying the entanglement distribution probability (EDP), the entanglement swapping probability (ESP), and the edge capacity (in terms of quantum channels) in several square-grid quantum network topologies. In all scenarios considered, the proposed PEP model consistently outperforms the LEP model in terms of the blocking ratio (*BR*). The superior performance of the PEP model is due to its ability to compute the number of purification rounds at the path level, thereby avoiding the redundant purification operations often demanded by the link-based model, while still maintaining the E2E fidelity threshold. This results in a reduced *BR* value by allowing a higher number of requests to be accommodated with the same network capacity. Moreover, the results reveal that at lower values of EDP and ESP (around 0.6), ESP has a stronger influence on *BR* compared to EDP, when other parameters remain constant. In contrast, when ESP is high (approximately 1), its impact on *BR* becomes less significant compared to the same range of EDP values. Overall, the results demonstrate that joint optimization of fidelity-aware online routing, purification planning, and resource allocation at the path level yields significant improvements in the blocking ratio under noisy intermediate-scale quantum (NISQ) constraints.

In future work, we plan to extend this study by considering the heterogeneous quantum network in terms of noise model and corresponding purification operations. We will consider the asymmetric purification model and heterogeneous decoherence times as well. This consideration results uneven distribution of Bell pairs across the edges of the quantum networks and leads to adopt mathematical models like the continuous-time Markov chain or the lumpability-based approaches for Bell pair distribution. Furthermore, in addition to the blocking ratio, we will also analyze quality of service (QoS) parameters such as delay and throughput, focusing on their joint optimization.

## Data Availability

The codes used and/or analyzed during the current study can be reached by following the GitHub link: https://doi.org/10.5281/zenodo.21869539.

## References

[1] Preskill, J. Quantum computing in the nisq era and beyond. *Quantum* **2**, 79 (2018).

[2] Bahrani, S., Razavi, M. & Salehi, J. A. Wavelength assignment in hybrid quantum-classical networks. *Sci. Rep.* **8**, 3456 (2018). 29472581 10.1038/s41598-018-21418-6 PMC5823948

[3] Cacciapuoti, A. S. et al. Quantum internet: Networking challenges in distributed quantum computing. *IEEE Netw.* **34**, 137–143 (2019).

[4] Komar, P. et al. A quantum network of clocks. *Nat. Phys.* **10**, 582–587 (2014).

[5] Li, Z. Swapping-based entanglement routing design for congestion mitigation in quantum networks. *IEEE Trans. Netw. Serv. Manag.* 10.1109/tnsm.2023.3275815 (2023).

[6] Li, Z. et al. Entanglement-assisted quantum networks: Mechanics, enabling technologies, challenges, and research directions. *IEEE Commun. Surv. Tutor.* 10.1109/comst.2023.3294240 (2023).

[7] Halder, J., Matus, E. & Fettweis, G. On the concurrent multipath entanglement distribution in quantum networks. In *GLOBECOM 2024-2024 IEEE Global Communications Conference*, 2791–2796 (IEEE, 2024).

[8] Cacciapuoti, A. S., Caleffi, M., Van Meter, R. & Hanzo, L. When entanglement meets classical communications: Quantum teleportation for the quantum internet. *IEEE Trans. Commun.* **68**, 3808–3833 (2020).

[9] Zhang, L. & Liu, Q. Concurrent multipath quantum entanglement routing based on segment routing in quantum hybrid networks. *Quantum Inf. Process.* **22**, 148 (2023).

[10] Halder, J., Rajabov, A., Bassoli, R., Fitzek, F. H. & Fettweis, G. P. Optimal routing and end-to-end entanglement distribution in quantum networks. *Sci. Rep.* **14**, 19262 (2024). 39164396 10.1038/s41598-024-70114-1 PMC11336113

[11] Van Meter, R., Satoh, T., Ladd, T. D., Munro, W. J. & Nemoto, K. Path selection for quantum repeater networks. *Network. Sci.* **3**, 82–95 (2013).

[12] Cuomo, D., Caleffi, M. & Cacciapuoti, A. S. Towards a distributed quantum computing ecosystem. *IET Quantum Commun.* **1**, 3–8 (2020).

[13] Sheng, Y.-B., Long, G. L. & Deng, F.-G. One-step deterministic multipartite entanglement purification with linear optics. *Phys. Lett. A* **376**, 314–319 (2012).

[14] Cicconetti, C., Conti, M. & Passarella, A. Resource allocation in quantum networks for distributed quantum computing. *In 2022 IEEE International Conference on Smart Computing (SMARTCOMP)* 124–132 (IEEE, 2022).

[15] Sutcliffe, E. & Beghelli, A. Multi-user entanglement distribution in quantum networks using multipath routing. *IEEE Trans. Quantum Eng.* 10.1109/tqe.2023.3329714 (2023).

[16] Pant, M. Routing entanglement in the quantum internet. *npj Quantum Inf.* **5**, 25 (2019).

[17] Chakraborty, K., Elkouss, D., Rijsman, B. & Wehner, S. Entanglement distribution in a quantum network: A multicommodity flow-based approach. *IEEE Trans. Quantum Eng.* **1**, 1–21 (2020).

[18] Zhou, L., Zhong, W. & Sheng, Y.-B. Purification of the residual entanglement. *Optics Express* **28**, 2291–2301 (2020). 32121922 10.1364/OE.383499

[19] Bennett, C. H. et al. Purification of noisy entanglement and faithful teleportation via noisy channels. *Phys. Rev. Lett.* **76**, 722 (1996). 10061534 10.1103/PhysRevLett.76.722

[20] Hu, X.-M. et al. Long-distance entanglement purification for quantum communication. *Phys. Rev. Lett.* **126**, 010503 (2021). 33480791 10.1103/PhysRevLett.126.010503

[21] Yan, P.-S., Zhou, L., Zhong, W. & Sheng, Y.-B. Advances in quantum entanglement purification. *Science China Physics, Mechanics & Astronomy* **66**, 250301 (2023).

[22] Chen, L. & Jia, Z. On optimum entanglement purification scheduling in quantum networks. *IEEE J. Sel. Areas Commun.* 10.1109/JSAC.2024.3380080 (2024).

[23] Wang, Z. An efficient scheduling scheme of swapping and purification operations for end-to-end entanglement distribution in quantum networks. *IEEE Trans. Netw. Sci. Eng.* 10.1109/TNSE.2023.3299177 (2023).

[24] Fan-Yuan, G.-J. et al. Robust and adaptable quantum key distribution network without trusted nodes. *Optica* **9**, 812–823 (2022).

[25] Wang, K. & Tan, C. W. Reverse engineering segment routing policies and link costs with inverse reinforcement learning and EM. *IEEE Trans. Mach. Learn. Commun. Netw.* 10.1109/TMLCN.2025.3598739 (2025).

[26] Xiong, N., Kuang, C., Liang, W., Chen, L. & Shen, M. Efficient routing scheme based on segmentation in quantum networks. *IEEE Internet Things J.* 10.1109/JIOT.2025.3612447 (2025).

[27] Wang, M., Tan, C. W., Xu, W. & Tang, A. Cost of not splitting in routing: Characterization and estimation. *IEEE/ACM Trans. Netw.* **19**, 1849–1859 (2011).

[28] Halder, J., Das, S., Paira, S., Chatterjee, M. & Bhattacharya, U. A multipath-based survivability scheme in energy-efficient eon. *IEEE Commun. Lett.* **22**, 2024–2027 (2018).

[29] Halder, J., Matus, E. & Fettweis, G. On the concurrent multipath entanglement distribution in quantum networks. *In GLOBECOM 2024-2024 IEEE Global Communications Conference* 2791–2796 (IEEE, 2024).

[30] Chen, L. et al. Cross-layer congestion control, routing and scheduling design in ad hoc wireless networks. *In Infocom* (2006).

[31] Van Meter, R. *Quantum networking* (John Wiley & Sons, 2014).

[32] Marin, A. & Rossi, S. On the relations between markov chain lumpability and reversibility. *Acta Informatica* **54**, 447–485 (2017).

[33] Vasan, V., Nico-Katz, A., Bash, B. A., Kilper, D. C. & Ruffini, M. Entanglement purification with finite latency classical communication in quantum networks. arXiv preprint arXiv:2509.03667 (2025).

[34] Dahlberg, A. A link layer protocol for quantum networks. In *Proceedings of the ACM special interest group on data communication* 159–173 (2019).

[35] Yen, J. Y. Finding the k shortest loopless paths in a network. *Manage. Sci.* **17**, 712–716 (1971).

[36] CPLEX. Cplex optimizer high-performance mathematical programming solver for linear programming, mixed integer programming, and quadratic programming. https://www.ibm.com/in-en/products/ilog-cplex-optimization-studio (2023).

